# Some Diseases of the Male Genital System

**Published:** 1908-01-04

**Authors:** 


### SOME DISEASES OF THE MALE GENITAL SYSTEM.

### III.—THE TREATMENT OF VAGINAL HYDROCELE.

This is either palliative or radical. Palliative treatment consists of periodically removing the contents of the hydrocele with a trocar and canula. In the pre-antiseptic days of surgery this was practically the only known form of treatment, for the reason that surgeons did not dare to perform an open operation in this situation. In view of the excellent results which are now obtained from operative treatment and also of the fact that the hydrocele refills after tapping in a period of time which varies, roughly speaking, from three to six months, palliative treatment is gradually falling into disuse. Inasmuch, however, as there are patients who refuse radical treatment, or whose general condition contraindicates operation, its technique will be described shortly. The patient is seated on the edge of a chair in front of the surgeon, who himself occupies another one. It is better to have the patient sitting down for two reasons: (i.) In case, as sometimes happens, he faints from the pain of the puncture, and (ii.) he is unable to draw himself away from the surgeon when he feels the point of the trocar. The scrotum is now grasped with the left hand, and the skin over it made tense, while the right hand holds the trocar, the end of the right forefinger being held on the trocar about one inch from the point, to prevent the instrument being pushed in too far. A position on the surface is chosen which is free from veins, and the trocar is pushed in rapidly in a direction backwards and slightly upwards. The trocar itself is now withdrawn, leaving the canula in position, and through this all the fluid drains away. When the hydrocele is quite empty, the canula is withdrawn, and the site of the puncture covered over with a small piece of gauze and collodion. The scrotum should be supported for a day or two with a suspender or a bandage. Asepsis is, of course, essential, otherwise cellulitis or gangrene of the scrotum and testis may result. Another important point is that the position of the testis must be accurately defined before tapping; it sometimes lies in front of, instead of behind the hydrocele, as in a normal case, and the results of driving the trocar into the testis are sickening pain and, possibly, the supervention of a hæmatocele.

#### Results of Injection Method.

This method hardly ever leads to a permanent cure of the hydrocele; very few such authentic cases have been recorded in the annals of surgery and other methods have therefore been introduced. The commonest is the introduction into the sac of the hydrocele, after the removal of the fluid with a trocar and canula, of irritant fluid which causes a low form of inflammation of the sac wall so that its surfaces adhere together. The fluid relied on to produce this result is either iodine or carbolic acid. If iodine is used the hydrocele should be completely emptied, exactly as in the palliative operation, and before the canula is withdrawn from two to four drachms of the Edinburgh tincture of iodine is inserted with a syringe. The scrotum is then manipulated so that the iodine comes into contact with the entire tunica vaginalis. After this about half the iodine is allowed to escape, the canula is carefully withdrawn, and gauze and collodion applied. The reaction to the fluid generally occurs in a few hours, and it is well to keep the patient in bed with the scrotum suspended for a day or two. After about four days the inflammation begins to subside, and the patient may begin to walk about; but the scrotum should be strapped for two or three weeks. At the end of this time the inflammation should have subsided completely. Carbolic acid is employed in exactly the same way, from half to one drachm of glycerine which is saturated with carbolic acid being injected. It seems to have some advantages over iodine in that it is more certain in its action and takes a shorter time to produce the required result.

#### Hydrocele in Children.

These methods must never be used in small children with hydrocele. In the first place the hydrocele is nearly always of the congenital variety, and communicates with the peritoneum, although the opening may be so small that the fluid cannot be reduced by manipulation. The proof of this is that if the child lies up the hydrocele often tends to disappear spontaneously; and, secondly, the tissues are unable to stand strong irritants as are those of adults, and there is a great danger of sloughing of the whole scrotum if they are employed. Hydrocele in an infant can very often be cured, as has been said, by keeping the child on his back and applying an evaporating lotion. If this fails the hydrocele should be punctured in a number of places with a sterilised needle, so that the fluid can run away into the tissues of the scrotum. If it recurs, recourse should be had to an open operation, in which the sac of the hydrocele is dissected out and removed.

#### The Radical Cure by Open Operation.

The ideal form of treatment for vaginal hydrocele which is now slowly but surely supplanting all other methods is the removal of the whole parietal portion of the tunica by open operation. An incision is made over the scrotum in its upper part and the fluid removed by trocar and canula. The testis and collapsed hydrocele can now be delivered out of quite a small wound. The sac of the hydrocele is opened with scissors, and the whole of the parietal portion removed by cutting it round quite close to the testis. A large number of small vessels bleed in the cut edge, and these must be carefully tied because it is important that all hæmorrhage should be arrested before the testis is returned to the scrotum. It is impossible that there should be any recurrence after this operation, because the whole wall of the sac is removed. This operation is much preferable to incising the tunica and then completely inverting it round the testis and bringing its edges together with catgut, or to partial excision of the parietal layer and union of the portions left.

